# A Case of Inflammation on the Glutei Muscles, &c. of a Threatening Tendency, and Accompanied with Violent Symptoms of General Irritation, Speedily Repressed and Cured by the Combined Internal and External Use of Cold Water

**Published:** 1799-10

**Authors:** Robert Kinglake


### *A Case of Inflammation on the Glutei Muscles, &c. of a threatening tendency, and accompanied with violent symptoms of general irritation, speedily repressed and cured by the combined internal and external use of cold water:—Communicated by* ROBERT KINGLAKE, *M. D.*

WERE the subsequent case of external inflammation, rapidly subdued by the salutary influence of reduced temperature, a solitary instance of its curative agency, and did it not form a series of similar, though less striking occurrences in my experience (which on some future occasion will probably be detailed to the public), it would have less claim to be rescued from oblivion, and be less worthy of record in your practical collection, than from this consideration it would seem to merit.

Mr. B. aged about forty, and of an athletic constitution, was attacked, in the beginning of August, with rigor, succeeded by febrile symptoms, a sense of preternatural heat, throbbing, and visible tumefaction, occupying the glutei muscles, and extending to the anus, rectum, perinæum, and membranous part of the urethra. The inflammation had been progressively increasing forty-eight hours before I saw the patient: at that time the irritation of the system was extremely violent, pulse hard, full, and rapid, insatiable thirst, skin hot and dry, partial suppression of urine, constipation, incessant, painful, and fruitless efforts to empty the bladder and rectum, considerable tension of the lower region of the abdomen, &c.

The local irritation, and, consequently, the general symptoms excited by it, had been much aggravated by the injudicious application of warm cataplasms to the parts affected, the internal use of terebinthinate medicines, oppressive weight weight of bed-clothes, unventilated room, &c. This noxious plan of treatment was by my direction reversed; the rigid discontinuance of whatever might inordinately excite, was enjoined; folded cloths, moistened in cold spring water were ordered to be applied to the inflamed parts, comprising the membra genitalia externa and abdomen, and to be renewed every half-hour or oftener, if a sense of unusual or inflammatory heat should sooner return.— To co-operate with this refrigerating plan, the patient was directed to dilute plentifully with cold water, drank in small quantities, at short intervals, and to persist in it as long as it proved grateful, or was demanded by febrile heat and thirst. The intestines and urinary bladder were copiously evacuated by divided doses of an aqueous solution of vitriolated soda, repeated, as long as necessary, at short intervals.

Immediate alleviation was afforded by the combined external and internal use of cold water, but the morbid sensations of the patient warranted its unremitted application for twelve hours, when the swelling, tension, and pain were so much diminished, as to admit of lengthening the intervals of its renewal. The succeeding forty-eight hours were employed in furthering the cure, by adapting the force of the reduced temperature to the decreasing influence of morbid heat, and on the third day inclusive from the commencement of this plan of treatment, the patient had no other remains of inflammation, than slight soreness of the part affected when pressed, and a livid hue on the surface. The general health, which suffered only from sympathetic irritation, also returned to its natural standard, and neither local nor constitutional inconvenience has since been experienced.

The salutary effects of refrigeration in attempering and correcting morbid heat, and obviating its probably suppurative, and possibly gangrenous consequences, were in this case very apparent. Had it been applied in less force, or uncombined with plentiful internal dilution, it most likely would not have proved equally effectual.

Farther experience will, perhaps, evince, that no principle of curing diseases is better founded, than that of combating redundant heat with proportionate cold, or, more pathologically speaking, of retrieving, by transferring media, the vitiated processes of organic action, which generate an undue and destructive temperature: nor are there, perhaps, any medicinal agents so uniformly operative, and so commensurate with the object to be attained, as cold water.

The patient's sensations afford a good practical rule in the application of reduced reduced temperature: when permanent chillineſs is not induced, its operation can have no hurtful tendency, and *vice verſa*.

The converſe of the propoſition, with regard to cold being ſalutary in repreſſing morbid heat, holds with reſpect to heat being beneficial in diſeaſes of deficient temperature, ſuch as febrile chills, paralytic affections, &c. and it will be accordingly found, that water, at the temperature of 100 degrees of Fahrenheit's thermometer, internally and externally employed, will, in ſuitable circumſtances, prove the moſt efficient, durable, and congenial ſtimulant.

